# Water fetching and musculoskeletal health across the life-course in Sub-Saharan Africa: A scoping review

**DOI:** 10.1371/journal.pgph.0003630

**Published:** 2024-09-03

**Authors:** Soren Meeuwisse, Susan J. Elliott, Alexa Bennett, Videsh Kapoor

**Affiliations:** 1 Southern Medical Program, University of British Columbia, Kelowna, British Columbia, Canada; 2 Department of Geography and Environmental Management, University of Waterloo, Waterloo, Ontario, Canada; 3 Department of Family Medicine, University of British Columbia, Vancouver, British Columbia, Canada; University of Groningen, NETHERLANDS, KINGDOM OF THE

## Abstract

The world is experiencing a global water crisis and Sub-Saharan Africa (SSA) is expected to be a hotspot for increasing global water scarcity in years to come. Water is quintessentially a gendered issue; indeed, sociocultural norms hold women responsible for household water collection, requiring them to travel far distances while carrying water. This paper reports the findings of a scoping review of peer-reviewed and grey literature that examines the relationship between water fetching and the musculoskeletal (MSK) health of women in SSA. The work is informed by a gendered life-course perspective, and the authors follow the PRISMA-ScR guidelines. Results indicate a bidirectional relationship between water fetching and poor MSK health as chronic and acute incidences of water carrying are highly related to MSK pain and dysfunction. This has negative implications for the overall health and wellbeing of women and their households. Gaps in the literature are identified, including the experiences of elderly people and people with various vulnerabilities. Recommendations from the literature are compiled to outline potential avenues of future research and innovation to better support the MSK health of water fetchers in SSA.

## Introduction

The population of Sub-Saharan Africa (SSA) is being faced with a combination of environmental, demographic, and social challenges that ultimately impact the musculoskeletal (MSK) health of women. Compared to the rest of the world, SSA countries experience high water insecurity and are expected to be the ‘hot spot’ of global water insecurity in years to come [1–4]. Water accessibility in SSA shows significant variation between countries and between rural and urban areas. Only 39% of the population has water connected to their homes, with this percentage dropping to just 19% in rural areas [5]. This water insecurity forces millions of women in rural and low-income communities of SSA to fetch and carry water daily [6] which can cause accelerated degenerative changes in their bodies [7–9]. There is a massive and unequal burden of MSK disease in SSA compared to the rest of the world [10], with women in SSA reporting a greater prevalence of mobility disability compared to men [11]. Due to sociocultural norms, women and female children are disproportionately impacted by water insecurity as they must assume the responsibility for collecting water for their households [6,12–14]. Many women’s daily lives revolve around water collection, as walking to and from water points and waiting in long queues consumes several hours of the day [15,16]. Water is quintessentially a gendered issue [17]; therefore, the United Nations calls for the prioritization of gender in water, sanitation, and hygiene (WaSH) reporting, policies, and agendas [18].

Concomitantly, the elderly population in SSA is expected to increase significantly over the coming decades, increasing from 31.9 million in 2019 to 101.4 million in 2050 [19]. An age-sensitive approach is crucial to accurately depict the complex and evolving challenges of water fetching at different life stages, throughout a lifespan, and across generations. A research perspective that is considerate of age is pivotal to formulating healthy growth, development, and aging strategies for the people of SSA as they face increasing water insecurity in years to come.

In 2018, Jo-Anne Geere and colleagues conducted the first systematic review of the impacts of water carrying on the water carrier’s overall health, which examined the physical, social, and mental health of water carriers around the world [20]. However, the relationship specifically between MSK health and water fetching has historically been neglected in academic research. Several recent studies attribute the consequences of water fetching to the global burden of MSK diseases, yet systematic and robust documentation of this phenomenon is lacking [20,21]. Building on the findings of Geere et al.’s systematic review [20], we first summarize their findings specifically related to MSK health in SSA, and then provide an update of the literature between 2017–2023. Further, few studies have explored the impacts of water insecurity through a life-course perspective [16,22,23], and these studies mainly focus on *non*-MSK health implications (ie. psychosocial health, overall wellbeing) in select countries (India and Kenya). The need for a life-course perspective [24] in the examination of water insecurity in low- and middle-income countries [LMIC] has been suggested in the literature, and this paper is the first to explore the relationship between water insecurity and MSK health in SSA through a gendered life-course perspective.

This paper aims to answer the following exploratory research question: What is the relationship between water fetching and the MSK health of females in SSA? This question involves a bi-directional exploration of the potential impact of water fetching on MSK health and the potential impact of MSK health on water fetching. First, we present the results within tables and narratively summarize themes according to age categories (children, adult women, and elderly women). Second, we discuss the implications of our findings with a particular focus on innovations and future research needed to address gaps in the literature and healthcare provision.

This paper contributes to our understanding of the progress towards achieving Sustainable Development Goal (SDG) 3 (Good Health & Well-Being), SDG 6 (Safe Water & Sanitization), and SDG 5 (Gender Equity). This research also contributes to the message advocated by Ma et al. [25] that injury prevention must be more properly recognized in the SDG agenda and included in the broad sector and stakeholder interests.

## Methods

Three databases were searched to identify potentially relevant peer-reviewed and grey literature articles, including MEDINE (Ovid), EMBASE (Ovid), and CINHAL (EBSCO). Hand-searching was used to supplement our database searches, and as a result, three included papers were identified that are not indexed in the listed medical databases [26–28]. Grey literature was systematically searched, following the methodology outlined by Godin et al. [29], to identify articles from targeted websites, grey literature databases, search engines, and field experts. The detailed search strategy can be found in the Supporting Information section of this paper (see S1–S7 Tables). Health Science librarians from the University of British Columbia and McMaster University were closely consulted for the database and grey literature searching strategies, and the database search strategy was adapted from the search protocol of Geere et al.’s systematic review [20]. The Preferred Reporting Items for Systematic Reviews and Meta-Analyses extension for Scoping Reviews (PRISMA-ScR) Checklist [30] and the Joanna Briggs Institute (JBI) Scoping Review Protocol [31] were resources used in structuring, conducting, and presenting this research.

Papers were included if they discussed MSK health (functioning, pain, disability, etc.) of people who fetch water for domestic purposes in SSA and were published in English. The type of negative MSK health outcomes analyzed in this review are preventable and related to the heavy load of carrying water or injuries experienced along the water fetching route due to environmental hazards (e.g. slippery terrain). MSK outcomes not analyzed in this review are related to genetic disorders, infectious diseases, or other health risk factors. Papers were excluded if they discuss health implications of water insecurity that do not impact the MSK system, do not impact the carrier of the water, or are not related to household water usage. Further, papers were excluded if MSK health was influenced by factors other than the physical loading of water and risks of walking paths (ie. sexual and gender-based violence, fights at water point queues, poor water quality, etc.). Despite the complex diversity across the large geographic area and between populations within SSA, the context of the included studies was limited to SSA to shed light on the females in this region with specific sociocultural norms, climate change impacts, demographic trends, MSK disability rates, natural resource availability, and economic states that may differ from other areas of the world. The inclusion dates of January 1, 2017, to current (March 15, 2023) were chosen to build on the systematic review findings presented by Geere et al. [20] which presents sources published between inception to 2017.

Both the primary investigator and an additional editor screened the titles/abstracts and completed full-text reviews for inclusion through Covidence. Data charting (Tables 1 and 2) and relevancy rating (within Table 2) were completed by the primary investigator and reviewed by all co-authors. No studies were excluded due to methodological quality; however, each article was assigned a relevancy rating by the primary investigator based on the methodologies and relevancy to this scoping review’s inclusion criteria and primary research question. High relevancy reflects that the article’s main purpose was to explore the relationship between MSK health and water carrying, and the study design and methodologies reflected this aim. Articles of medium relevancy include significant information on the MSK health of water carriers (which must be within the abstract results), but the main research purpose is not the link between MSK health and water fetching. Low relevancy means that the article provides some novel data or insight on the MSK health of water carriers, but it only has minimal, vague, anecdotal, or very general information. Articles of medium and low relevancy were included because, despite their relatively weaker data and less compatible methodologies for this review, sections of their results and discussion do contribute to the limited literature available on the MSK health of water fetchers. Three papers were assigned for high relevancy, three for medium relevancy, and six for low relevancy.

**Table 1:** Summarizing Geere et al.’s systematic review conclusions related specifically to MSK health in SSA populations (2018).

Author	Objective	Experimental Design	Population/Samples	Key Findings	Dates
**Geere et al., 2018 [20]**	To explore relationship between water carriage and the water carrier’s health	Systematic review. Narrative results.	8 papers relevant to MSK health + SSA [12,32–38]	• Overall: strong qualitative and moderate quantitative evidence that carrying water is associated with pain and physical injury • Head, upper back, chest/rib, hands and abdomen/stomach pain is correlated with ‘axial compression’ (ie. head-loading) • Most common areas of reported pain: neck, back, chest • Both women and children report pain and accidental injuries related to water fetching pathway hazards • For people reporting pain, the location and type of pain was related to a chronic history of water carrying and the pain areas were correlated • The work of water carriage is more often associated with harm rather than benefit to the water carriers’ health.	Inception—Nov 2017

**Table 2:** Results of papers published 2017–2023 (n = 12).

Authors, Year (citation)	Objective	Experimental Design & Observation Methods	Population/Sample	Key Findings	Dates	Relevancy
**Assefa et al., 2021** [15]	To strengthen understanding of the relation between WaSH access, gender, and social inclusion.	Explorative qualitative. 21 KII and 9 FGD.	Oromia, Ethiopia. Urban & rural. N = 102 men, women, children; & local professional representatives. Purposive sampling.	• 20/25L jerry cans pose a great risk for MSK injury to women • People living with disability or HIV, the elderly, women, children, and people without donkeys/carts are disproportionately impacted by water fetching distances and accessibility • Children often carry more than physically capable, causing injuries	n/a	LOW
**Collins et al., 2019 [39]**	To explore: (1) experiences of water acquisition, prioritisation, and use. (2) consequences of water insecurity	Exploratory qualitative. Observational cohort study (Go-Along and thematic rankings; photovoice)	Nyanza, Kenya. Urban & rural. N = 40 women in an area of high HIV prevalence. Mean age: 28. Purposive sampling.	• Even during pregnancy and post-partum, women were responsible for water provision, which required walking on dangerous terrain and entering bodies of water to collect water. Slips, falls, and resulting injuries were reported as frequent. • Key MSK findings for maternal and child health: higher risk of injury for these populations • Reported consequences: back, abdomen, and chest pain; breathlessness; and fatigue.	Sept-Oct 2015	LOW
**Gebeyaw et al., 2021 [40]**	To explore the challenges faced by homeless, older, rural to urban migrants.	Cross-sectional study & qualitative descriptive case study. 10 in-depth interviews, 4 KII, and document review.	Kobo Town, Ethiopia. Urban. N = 10 [W = 3, M = 7] homeless (>1 yr), older (>60yrs) migrants. Mean age: 72. Purposive sampling.	• Elderly people experience a lack of physical strength, age-related disabilities, and mobility restrictions that impede their accessibility to water • Fetching water and going to the river for WaSH purposes are 2 of the 4 main activities for daily survival by participants.	n/a	LOW
**Giljam-Enright et al., 2020 [41]**	To highlight the sociocultural role performance of Xhosa women with stroke.	Collective case study. Quantitative data questionnaire and qualitative individual semi-structured interviews.	South Africa. Urban & rural. N = 19 Xhosa women with stroke history in low socioeconomic areas. Mean age: rural 49; urban 56. Purposive consecutive sampling with snowballing.	• Xhosa women who have experienced stroke were still responsible to fetch water, but they experienced many physical challenges due to stroke disability. • Only one rural participant was able to continue regular daily tasks, including water fetching, post-stroke due to the physicality of the role.	n/a	LOW
**Hawker et al., 2021 [42]**	To determine the prevalence and risk factors of back pain in a cohort of pregnant women.	Descriptive cohort. Quantitative: clinical record analysis, questionnaires (sociodemographic, pain rating).	KwaZulu-Natal, South Africa. N = 303 black South African pregnant women from an antenatal clinic in a resource-poor setting. Mean age: 26. Convenience sampling.	• Carrying water was the only ADL associated with LBP pre-pregnancy & at the first antenatal care visit • LBP was the most common MSK disorder experienced, and it was chronic in nature. • LBP prevalence was low, but the women with LBP had a reduced capacity to cope with daily life.	Oct 2015- Oct 2016	MEDIUM
**Hiestand-Saho et al., 2022 [43]**	To determine if neck pain or functional limitations could be explained by head-loading characteristics.	Cross-sectional study. Objective device measuring; subjective pain scale.	Gambia. Rural. N = 39 women. Age years: range 18–45, mean 33. Convenience sampling.	• Neck pain was most reported (N = 35) by head-loaders, but LBP (N = 14) was more associated with functional limitations. • Less head-loading weight was carried by women with moderate-severe pain and with experiences of functional limitation. • Weight of load, cervical range of motion, and proprioception can partially explain the functional limitations and pain experienced. • 11 women reported functional limitations that impaired ability to perform ADLs	Nov-Dec 2017	HIGH
**Kadota et al., 2020 [44]**	To assess the impact of heavy load carrying on MSK pain and disability among women.	Exploratory cross-sectional study. Quantitative structured surveys, anthropogenic measurements, weighing of loads carried, GPS measuring.	Shinyanga Region, Tanzania. Rural. N = 82 women. Mean age 32. Purposive sampling.	• Higher load-carrying exposure related to greater pain and disability, especially for LBP. • Increased odds of LBP and knee pain with greater weight, duration, and cumulative loading. • No significance for odds found for head, neck, or foot/ankle pain. • 51–90% of participants reporting pain related disability during wood, water, or other material collection • ~50% predictability of LBP within last year for an average load carried by women on 18.8kg	July-Aug 2016	HIGH
**Kamya et al., 2021 [45]**	To report children’s experience on water accessibility and utilization.	Cross-sectional mixed methods. Quantitative surveys and qualitative FGD.	Rakai district, Southwestern Uganda. Rural. Survey N = 405 and FGD N = 24 of school-aged children (11–17 years)	• Water fetching is the most common chore performed by children and the majority of children report it to have a direct threat on their wellbeing due to long distances to and risks at the water point. • Aches, pains, and the perception of head-loading compromising physical growth are negative health impacts of water carrying frequently reported by children. • Children play a central role in household water provision and fetching (more than adults).	n/a	MEDIUM
**Osinuga et al., 2022 [46]**	To examine the relationship between domestic work, sociodemographic characteristics, and MSK pain symptoms and severity	Cross-sectional study. Interviewer-administered survey data.	Ibadan, Nigeria. Rural. N = 356 women. Age years: range 18–45; mean 31. Purposive sampling.	• Frequently fetching water over long distances and time is associated with higher odds of LBP and neck/shoulder pain. • 58% of women experienced LBP, 30% experienced neck/shoulder pain, and 29% experienced elbow/hand/wrist pain. • ~1/3 of all women did not experience MSK pain in any body region, ~1/3 experienced pain in one region, 20% in two body regions, and 14% in all three body regions.	n/a	MEDIUM
**Udofia & Oloruntoba, 2019 [27]**	To explore the accessibility to potable water in homes for the disabled in Nigeria.	Cross-sectional. Mixed methods: questionnaires, FGD, and KII.	Ibadan, Nigeria. N = 64 physically challenged people living in homes for people with disabilities. Age years: range 8–38; mean 20. Purposive sampling.	• 23% of physically disabled respondents fetch water themselves (n = 15); 77% rely on someone else at the facility (caregiver or friend who is not physically disabled) to fetch water • Reported pain experienced by physically disabled persons when fetching water themselves: back pain (n = 7), chest pain (n = 3), leg/hand pain (n = 5).	n/a	LOW
**Venkataramanan et al., 2020 [21]**	To describe the frequency, characteristics, and correlates of water-fetching injuries.	Cross-sectional survey. Primarily quantitative, some qualitative.	24 sites in 21 LMICs; 8 SSA countries included. N = 6291 households. Simple random sampling. Mean age 38.	• 85% respondents reported 1 or more past experiences with water-fetching injuries, and 13% reported current prevalence • Water fetching injuries are significantly correlated with being female, higher household water insecurity scores, rural or periurban residence, use of off-premise water sources, and increased time spent collecting water. • Common elements of injuries: **(1)** Type: Fractures and dislocations (29.2%), pain (22.2%) **(2)** Bodily location: lower limbs (61.1%) **(3)** Context: dangerous terrain (69%), poor roads (24%), weather (heat/rain) (7%) **(4)** Mechanism: falling (76%) • 2 of the 5 LMICs with the greatest proportion of participants reporting water fetching injuries were SSA countries (Kenya; Ghana)	2017–2018	HIGH
**Winter et al., 2019 [47]**	To examine correlation between WaSH accessibility and health-related quality of life.	Cross-sectional quantitative survey.	Mathare slum, Nairobi, Kenya. Urban. N = 552 women. Age years: range 18–75; mean 35. Random sampling for households; Kish methodology for women.	• No significant association between women’s WaSH source accessibility and needs and their self-perceived physical health outcomes after adjusting for socioeconomic covariates.	2018	LOW

**ADL**: Activity of daily living.

**GPS**: Global Positioning System.

**KII**: Key Informant Interview.

**FGD**: Focus Group Discussion.

**n/a:** Not available.

**[W = #, M = #]:** Number of women and men included in sample.

## Results

**Fig 1:**
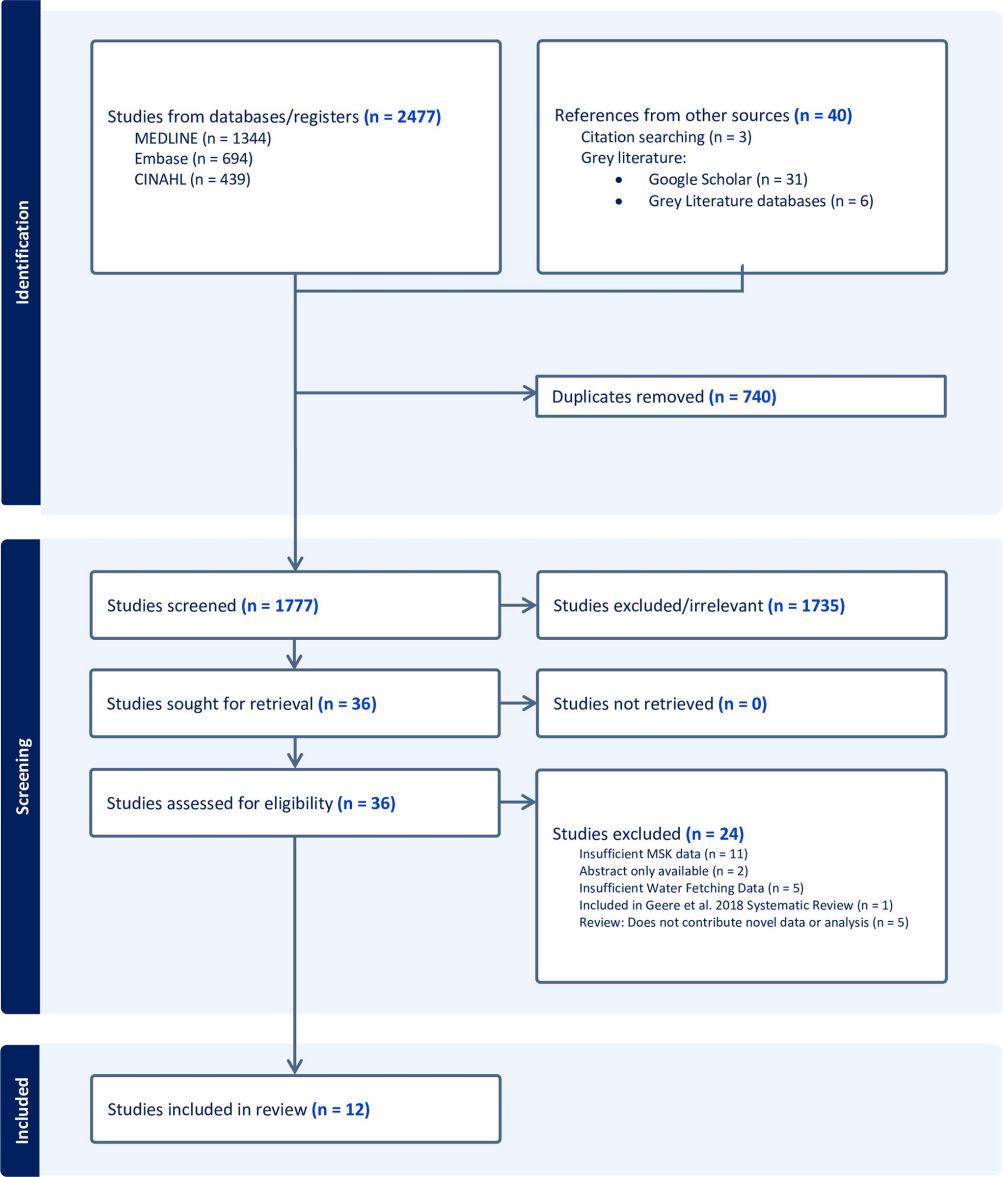
PRISMA flow diagram.

The process of searching, screening, and selecting included articles can be reviewed in the PRISMA Flow Diagram (**Fig 1**). Peer-reviewed database and grey literature searching retrieved 2,517 articles. After the removal of duplicates, 1,777 articles were screened based on title and abstract and 38 moved onto the full-text review. Twenty studies were included in this scoping review; the 8 articles included in Geere et al.’s [20] systematic review that are relevant to MSK health and SSA are summarized in **Table 1**, and the 12 included papers published thereafter (2017–2023) are charted in **Table 2**. A summary of the common themes that arose in the results is outlined in **Table 3**.

**Table 3:** Summary of common themes.

1.There is a bidirectional relationship between water fetching and poor MSK health: water fetching is much more associated with harm rather than benefit to the water carrier’s MSK health, and poor MSK health adversely impacts the ability to fetch water.
2. Water carriers commonly experience chronic MSK pain, specifically in the low back and neck, but also in the upper and lower limbs and chest.
3. Women and children are primarily responsible for water fetching, with men only collecting water in rare or extreme circumstances.
4. Deeply ingrained sociocultural gender norms uphold females’ primary responsibility for household water provision, despite intersecting vulnerabilities that expose women to additional challenges.
5. A life course perspective has very limited research. Most studies focus on female children and adults with limited literature focusing on elderly women, and no studies applied a life-course perspective.
6. Acute accidental injuries due to slips and falls on risky water-fetching terrain are common for all age groups.
7. Head-loading is by far the most common method of water carrying.
8. Rural populations experience greater water-fetching physical challenges, pain, and injury compared to urban populations.
9. Poor MSK health of the water carrier has diverse negative implications for the overall well-being of that individual and their household.
10. There are large gaps in the literature regarding the MSK health of water fetchers in SSA.

These results indicate that water fetching is associated with poor MSK health that is chronic in nature due to habitual loading and acute in nature due to environmental hazard-related injuries while walking with heavy loads. It was found that water fetching influences MSK health, and in turn, MSK health influences the capability to fetch water. Women and children are primarily responsible for the task of water collection and commonly report poor MSK health related to water fetching. MSK consequences are most commonly reported in the low back, neck, chest, and upper and lower limbs. There are minor inconsistencies between studies on which area of the body most commonly experiences pain or injury, and these inconsistencies could be attributed to a) the population or age of study participants, b) differences in water fetching methods, distance, or tools, c) inconsistencies in the way in which MSK pain is defined, conceptualized, and measured in studies, or d) study design. One study found no significant correlation between water accessibility and reported physical outcomes [47]. However, this study noted that their findings are surprising, inconsistent with previous literature, and can be explained by the population sample and limitations of the study methodology [47].

No papers concluded that water fetching had an overall positive impact on MSK health. However, women and children do report some indirect benefits of water fetching. For example, water fetching permits positive socializing opportunities for women and children in their daily lives [45,48]. Further, women and children report feeling pride for carrying large amounts of water, which proves themselves to be suitable as a wife and provides a sense of self-worth and purpose. Thus, women and children often overload their water buckets with more than they can comfortably carry [12,41,49,50].

Two patterns of pain distribution were identified due to chronic loading. First, ‘axial compression’ due to head-loading is strongly associated with head, chest/rib, upper back, abdomen/stomach, and hand pain [20]. Second, ‘soft tissue strain’ is slightly associated with upper limb, low back, neck, and lower limb pain [20]. Geere et al. report that people who head-load have a much higher prevalence of pain compared to people who carry water by other means, and, unsurprisingly, people who do not carry water report a lower prevalence of pain [20]. Due to compressive loading, it is hypothesized that tissue deformation and long-term structural changes of bone and soft tissue are caused by water carrying. Previous studies have related head-loading to arthritis [12,51], cervical spine degeneration and cervical spondylosis [7,9,20], and low back pain related to muscular strain and L4/L5 vertebrae joint torque [52]. These conditions are common in women in Africa [53] and introduce great risk for neurological impairment and disability [20,51]. Further, fatigue and water carriage are moderately quantitatively related and strongly qualitatively related, with fatigue increasing the risk of MSK injury [20]. Water fetching injuries are significantly correlated with being female, higher household water insecurity scores, rural or periurban residence, use of off-premise water sources, and increased time spent collecting water [21].

Additionally, acute MSK injuries can occur due to risky terrain and falling while water fetching. Water fetchers perform an obstacle course, maneuvering their bodies in risky manners while navigating ditches, roadside laundry and cooking, wet and slippery ground, sharp objects, narrow spaces, clotheslines, trees, rocks, and more [20,21,52,54,55]. Sharing a walking path with a road increases the risk of traffic-related injuries, slips and falls are common, trash and other urban objects introduce dangerous obstacles, and hilly terrain increases the physical strain [54,56]. Acute injuries are of significance because they can lead to the inability to fetch water or maladaptive compensatory MSK patterns while water fetching, facilitating chronic MSK pain and injury.

The methods of transporting water from the point of collection to the home vary depending on available resources and socioeconomic status. Most commonly used are 20/25L jerry cans or buckets, however, containers holding up to 60L are also used. These vessels are often carried by hand, back, or most commonly head-loading [12,20,51]. Wheelbarrows, bicycles, donkey carts, or paid labourers are used if these resources can be accessed or afforded [15,57].

Not only does water carrying have negative implications for the water carrier’s MSK health, but the water carrier’s experience of pain or disability impacts their ability to fetch water [20,27,41,43]. Water fetchers that report pain tend to walk shorter distances to water [20] and carry less water [43], risking the quality of the water source and reducing the amount of water accessed. It is common for women to assume the risk of collecting nearby poor-quality water instead of higher-quality water from a further water point, thereby attempting to reduce the physical burden and time spent collecting water [58]. These examples showcase how the risk of MSK disability may present a barrier to obtaining adequate quantities of water from a safe source. This point is well portrayed by Geere et al. [20] (p22): “Because water is essential for life, but fetching it is often not safe, water carriage is also a barrier to ensuring safe and inclusive societies, and decent work for all, a further challenge to reducing poverty in all its forms.”

### Life-course categories

#### Children

Six studies included in this review focus on children, defined as being under the age of 18, with five of these studies included in Geere et al.’s review [32,34,36–38] and one study published more recently [45]. Children from several SSA countries associate their high rates of neck and back pain with their participation in hazardous water carrying [32,34,36,38,45]. Female children report more pain than male children [36], and children are aware of the MSK risks of water fetching, including how this can impact their long-term physical health and ability to function in society [45]. Kamya et al. assert that children, more than adult women, play a central role in household water fetching as most children report carrying water 2–3 times every day, even on school days which requires fetching in early and late hours [45]. These researchers recognize that this finding is different from many other papers that claim adult women are primarily responsible for household water collection, and they claim that the ‘misconception’ that adult women bear the largest burden is due to the underrepresentation of the voices of children [45]. Whereas 83.4% of children carried water via head-loading in this study, only 10.1% used a bicycle, and the vast majority of the bicycle users were male children [45]. Bicycles, carts, or other devices may reduce time spent fetching or change the physical loading more favourably. Male children may have a more positive physical experience with water fetching compared to female children due to their gendered access to such devices [36,45,51].

#### Adult women

Seven included studies’ populations were adult women (>17 years old) [39,41–44,46,47] and five studies focused on the household level, which is non-specific in terms of age [12,15,21,27,35]. It is reported that the likelihood of MSK injury is 1.5 times greater for women water fetchers compared to male water fetchers in SSA [21]. Females may experience and report greater rates of MSK pain and injury compared to males for several reasons. First, females are socialized into gender roles that allocate primary responsibility for water collection; therefore, they are more exposed to the injury risks of water fetching [45]. Adult men rarely carry water, and it is often only men without a wife or family or with ill family members who fetch water [12,15,59]. Second, physiological differences, such as slender spines and less muscle mass, may predispose females to injury more than males [21]. Many areas of SSA have highly variable and unreliable water accessibility, therefore an uneven distribution of physical labour over time is often necessary to provide sufficient water to a household, increasing the risk of overloading, fatigue, pain, and injury [21,28]. ‘Maternal buffering’ can be defined as how women assume hardships to provide greater quantities of water for their families, which includes collecting water despite pain [28,39]. Sarkar [28](p217) explains that “[w]omen’s responsibility to fetch water for their household epitomises universal patriarchy, as it has been socially reinforced and culturally justified.”

The included studies also highlight complex vulnerabilities experienced by water fetchers, such as low socioeconomic status, motherhood, rural settings, people living with disability, people living with HIV, migrants, refugees, asylum seekers, stroke survivors, and pregnant and postpartum women. For instance, the adult stage of life includes the common child-bearing years; however, despite pregnancy and recent childbirth, women’s water collection responsibilities do not shift [39]. One study found that water carrying was the only self-reported factor associated with low back pain (LBP) pre-pregnancy and in the first trimester, and this chronic LBP impacted their ability to cope with daily life [42]. These findings are of significance because the SSA population has a high prevalence of pregnant women, and it is common for women to carry both water and a child over long distances, thus increasing their MSK strain. In a cohort of Tanzanian women water carriers with high rates of LBP and knee pain, 40% of the women had three to four children, an additional 40% had five or more children, and 64% had their first child as a teenager [44].

Furthermore, two included studies highlight the challenges introduced by physical disability. The assistance of family and friends was required to access enough water for physically challenged people living in homes for people with disabilities in Nigeria [41] and female stroke survivors in South Africa [27]. However, these research participants often also had to fetch water themselves which resulted in highly painful experiences and falling along the pathway. Other studies have reported that people with disabilities in LMICs experience discrimination, abuse, and stigma when using public water points [60,61], and that they face the least access to WaSH services [62]. Thus, while water fetching can cause physical disability, this physical disability in turn causes far-reaching physical, social, and mental health consequences. MSK dysfunction further exacerbates the vulnerabilities that lead to disability in the first place. It is a continuous cycle of water fetching causing reduced MSK health, and such health consequences further heighten an individual’s vulnerabilities, poor health status, and access to water. Living with a physical disability has broad negative implications on quality of life and economic stability in the short- and long-term for both an individual and their household [63,64].

#### Elderly

Research related to water fetching and MSK health in SSA has focused primarily on child and adult women with very minimal research on older populations. Only one of the included studies focused specifically on older people over the age of 60 years, however, this study was rated as low relevancy [40]. Therefore, there were no studies found specifically examining the relationship between MSK health and water fetching in elderly populations despite this age group commonly having to fetch water. The challenges faced by this population include reduced physical strength, mobility restrictions, and age-related disabilities that restrict their access to water for daily survival needs [40]. The lack of published literature focusing on elderly people is significant given the expected tripling of the senior population of SSA over the coming years [19]. While the population of SSA is comprised of proportionately more youth than other age groups, the absolute number of elderly people in SSA is extremely large and growing at a significant rate that will impact health systems and population needs.

Given their essential economic, familial, and community roles, the physical functioning of many grandparents in SSA is particularly important in their later years [65]. For instance, many parents in SSA have either succumbed to diseases, particularly HIV/AIDS, or migrated to urban areas in search of better economic opportunities, leaving grandparents responsible for tasks such as water fetching and childcare [66,67]. Skipped generation households, where grandparents care for their grandchildren in the absence of the middle generation, are on the rise in SSA [68]. This trend increases the reliance on the well-being of many elderly individuals in these regions. Daily water fetching is challenging, painful, or often not physically possible for seniors with existing MSK disabilities [65], and this task may cause or exacerbate injuries. As a result, grandchildren often have to fetch water during school hours to ensure household survival, which compromises their educational opportunities and has long-term impacts on their livelihoods and overall wellbeing [65,69]. There is a pressing need for increased social resources to support these grandparents’ physical functioning and overall health, and this support will yield significant benefits for both the family and society [19].

## Discussion

Safe and accessible water has been declared an essential human right by the United Nations (UN) [70], and the sixth SDG aims for universal access to drinking water and sanitation by 2030 [71]. This review outlines how women in SSA are resigned to the harmful MSK impacts of water fetching to achieve a fundamental human right of water accessibility. Short of the SDG target, water is neither reasonably safe nor accessible when considering this population’s physical health. MSK health, or the lack thereof, has large implications for individual and household overall well-being, especially in low-resource settings in SSA where livelihood is largely dependent on physical labour. Fetching water is an everyday experience for millions of women around the world, and this practice is predicted to persist for many years due to deeply ingrained sociocultural gender norms and increasing water insecurity, as a result of climate change and population growth [4,72].

Due to complex interactions between gender, culture, environment, identity, class, and race, the unequal responsibility and health outcomes associated with water carrying are often accepted by women as a norm [73]. Pain and injury are often not questioned and assumed to be a necessary, unavoidable part of life. Females may also under-report pain and associated risks since water fetching is a socialized expected duty. Therefore, unfavourable experiences may be invisible to females [51,55]. Further, due to unequal power relations between men and women, maintaining sociocultural roles is often perceived as more important than the physical pain experienced by women [73]. Women’s household labour and related health outcomes are largely unknown due to the lack of research on the MSK health implications of water fetching in SSA and around the world. This lack of information risks perpetuating gender inequity in one of the most dangerous ways: invisibility. Therefore, this review aims to bring greater attention to the health of females of all ages by sharing their voices and highlighting neglected areas of physical healthcare.

Water scarcity greatly influences numerous facets of life in Sub-Saharan Africa, impacting individuals and society as whole. On the individual level, there are many physical, psychological, and sociocultural health implications of water insecurity [39]. Specifically related to water fetching, these consequences include malnutrition and anemia [13]; diarrheal diseases [74]; the economic and educational opportunity cost [34,75–77]; intimate partner violence and sexual and gender-based violence [78–80]; drowning at water sources [45]; and psychological stress [15,28,81]. At the population level, water scarcity has profound public health consequences. These impacts include: exposure to waterborne diseases, reduced access to hygiene and sanitation, and malnutrition; all of which have the common result of increased risk of disease transmission and epidemics [82,83]. Healthcare systems rely on access to clean water and face increased costs associated with treating water-related diseases and addressing the long-term health consequences of water scarcity. The health of pregnant women and children is influenced by their increased susceptibility to infectious agents, with access to clean water being essential for adequate prenatal and postnatal care [84]. Families may spend considerable time and resources fetching water from distant sources, limiting income-generating activities and educational opportunities for children [75–77]. Competition over limited water resources may exacerbate existing tensions and conflicts within communities and between neighboring regions [85]. Water access drives population displacement, migration, and urbanization that may further heighten these tensions and contribute to living scenarios that increase risk of disease transmission and other health consequences [86–88]. Lastly, as highlighted in this paper, gender inequality is a public health issue which is exacerbated by water scarcity. As women hold the primary responsibility for water fetching, they experience gender disparities in health outcomes and opportunities for personal and economic development. It is essential to consider the wide range of consequences stemming from water scarcity. By identifying patterns of MSK dysfunction related to water fetching and addressing knowledge gaps, this paper aims to inform future research and MSK healthcare interventions to prevent, reduce, and rehabilitate poor MSK health, and ultimately contribute to improved individual and societal health outcomes.

### Innovations

Societal, cultural, behavioral, infrastructural, and ergonomic interventions must be strategized to reduce the burden of MSK pain and dysfunction in SSA. It is understood that women play a crucial role in household WaSH accessibility [89], yet women are largely excluded from water management, decision-making, and implementation processes which are often led by able-bodied men [15,90,91].

A transdisciplinary approach is required to address the complex nature of this issue. Many studies have suggested business or governmental policy models to improve water accessibility and reduce the need for carrying water, such as increased piped water. However, this is an unrealistic goal for millions of households for many years to come due to a combination of climate change, resource degradation, increasing populations, pollution, conflict, and gender inequity [15]. Further, water carrying is a complex sociocultural phenomenon with both positive and negative implications for women, children, and seniors. For now, water carrying exists and will persist due to strong sociocultural traditions tying women to this duty. Therefore, policies to improve water accessibility are crucial for the health and well-being of women and households in SSA.

Many authors have suggested policies to prevent such injustices or to alleviate suffering. These suggestions fall under the main categories of increased water accessibility [20], improved gender equity [21], public health messaging with the provision of water buckets [15,92], and improved healthcare accessibility. It has been suggested that there should be an increase in ergonomic aid availability, climate-resilient water-fetching tools, safer pathways, and public health messaging to promote male involvement in water fetching [21,93]. Additionally, there is a need for more non-communicable disease initiatives and water-related interventions to consider the link between MSK health and water accessibility [21,94].

Currently, available international WaSH indicators do not include factors of physical safety and accessibility [21]. Greater advocacy for local governments to improve WaSH accessibility is needed, particularly through a Gender and Social Inclusion lens to advocate for socially excluded groups and recognize social and gender inequality [15,16,27,40–42,44]. As water carrying and MSK disability are closely linked, the United Nations mission to “ensure equal access to persons with disabilities to clean water services” becomes highly relevant to water accessibility policies [95].

The WHO asserts that rehabilitation services are an essential component of universal health coverage [96]. More than 50% of the people who require rehabilitation services in LMICs do not have access to this essential service that helps them continue to live healthy, independent, and dignified lives, and COVID-19 largely reduced the provision and progress of such services [96]. There is minimal research on the causes of and healthcare solutions to MSK disorders in LMICs [44], and affordable context-specific MSK healthcare is required for the poor, vulnerable, and displaced people of SSA who fetch water daily [10]. SSA has an extreme lack of MSK healthcare services, workers, training programs, professional organizations, and regulating board requirements [97–100]. To support the water-fetching populations highlighted in this paper, MSK healthcare cannot be a luxury service available only to high-income countries or affluent populations, but rather a basic component of primary healthcare services available to the most vulnerable people.

A study on water fetching in India by Patil and Sangle [52] suggested the use of an innovative wheelbarrow-like device that reduces the torque on the L4/L5 joints and stress on the neck, trunk, and upper limbs. Vinay et al. [101], also in India, devised a backpack that reduced the time, distance, and energy of water fetching, thus increasing the quantity of water carried per trip. These technologies allow for postures that lower the risk of injury and pain and reduce energy utilized by the water fetcher compared to the traditional methods of head-loading. However, males are much more likely to have access to and use water-fetching aids such as bicycles and carts [36,45,51,91], indicating that regardless of the technology, males rather than females may have preferential access to other innovative devices. This may influence the real-world benefit of innovations introduced to water fetching communities. Further, even if a low-cost and sustainable innovation is successfully adopted in one community, that does not indicate transferable success in another community with different social, physical, economic, political, environmental, or intellectual infrastructures. There is no ‘magic bullet’ solution to improving the MSK health of water fetchers across the globe or within SSA countries. Water fetchers are the experts with local knowledge to lead ‘bottom-up’ innovative change [102–104].

While research and attempts to introduce innovations to assist water fetching in culturally safe manners can be beneficial, they may be short-term band-aid solutions to the greater and underlying issue of poor water accessibility. As water fetching is an integral part of life in SSA and will be for many years to come, such innovations of policies and technology may have the potential to help reduce the MSK health burden in SSA if they are prioritised by decision makers, developed with key stakeholder involvement for an inclusive design, thoroughly tested, and continuously re-evaluated.

### Future research recommendations

Future studies are warranted to better understand the relationship between water fetching and the water carrier’s MSK health, permitting assessment of the true burden of inadequate water accessibility. Studies that examine this relationship through a life-course perspective and with specific interest in older populations and people experiencing additional vulnerabilities are greatly needed to account for many different lived experiences and to address the gap in the literature identified by this review. We also suggest that future studies assess the impact of water fetching beyond one individual’s lifespan; or in other words, evaluate how water insecurity relates to intergenerational MSK trauma, or how MSK trauma relates to intergenerational water insecurity.

Several studies recommend that future research quantifies the relationship between MSK health and water fetching in larger and longer epidemiological studies [20,44], with the application of a validated pain scale [20,21], biomechanical technology, or a clinical assessment by a trained healthcare professional to reduce the reliance upon subjective self-reported pain, injury, and disability [20,44]. Validated pain scales or clinical assessments can increase the objectivity of MSK health outcomes and reduce inherent biases of self-reported MSK health, thus permitting the measurement of progress toward the SDGs and other global health goals. Further, future research has been suggested to explore the specific features of water-carrying MSK injuries, such as prevalence, severity, implications, injury type, mechanism, environmental context, and bodily location [21].

Lastly, it is suggested that future papers clearly outline how MSK pain, injury, and disability are defined, conceptualized, and measured; and there should be consensus across papers for appropriate comparison. There are significant inconsistencies across studies for how MSK pain, injury, disability, dysfunction, or disease are defined, conceptualized, and measured. This terminology inconsistency is more significant than a limitation of this review or included papers, but it is an issue of this field of research overall and deserves thorough attention.

Most articles do not clearly outline the differences, similarities, and boundaries between these various terms that are used across studies and interchangeably within studies. Since many papers include self-reported data from participants, these terms are influenced by the participants’ and researchers’ understanding, perceptions, and communication. This terminology inconsistency makes it challenging to assess the similarities, differences, and patterns between the papers. Only one paper explicitly outlined their definition criteria of ‘pain’ and ‘disability’ [44], and importantly, these definitions seem to be more exclusive than other studies’ criteria of ‘pain’ or ‘disability.’

Furthermore, pain and disability are complex experiences influenced by many biological, sociocultural, and psychological factors, such as genetics, expectations, and norms [105,106]. For instance, mental stress and fatigue influence self-reported pain intensity, and pain is a symptom of injury or disability [21]. For many studies, it is unclear at what point pain becomes injury, and injury becomes disability. Geere et al. [20](p7) note that using self-reported pain is “appropriate and necessary” because pain is a “subjective and emotional experience” and that pain-intensity scales are a reliable and valid method of assessing pain. However, all studies in Geere et al.’s systematic review include self-reported pain without a validated pain scale. Out of the 12 studies included in this scoping review, most used surveys or interviews to assess MSK pain and injury experiences, three assessed pain with a validated pain scale [42–44], and one used objective technology to measure range of motion and proprioception [43]. Therefore, terminology inconsistencies introduce challenges to judiciously understanding the MSK health reported in the included studies and to assess the overall message provided by the body of literature.

### Limitations of this scoping review

None of the included studies are longitudinal cohort designs so no causal or long-term relationships can be concluded about the MSK health of water fetchers. Therefore, the aim is to determine correlation rather than causation. Further, the quality of the included papers was not assessed, and no studies were excluded based on methodological quality.

The findings of this review are based upon many studies that neglect to account for the potential confounding impact of other types of load-carrying, such as firewood, or other types of habitual manual labour performed by the studied populations, such as cooking, cleaning, farming, and hawking. As such, confounding variables could be unaddressed in this review’s analyses. Two included studies account for other types of load-carrying, but they make no distinction between MSK pain related specifically to water fetching or other means [43,44]. A recent systematic review examined the combined health impacts of both water and solid fuel carrying around the world and concluded that women are most responsible for such transportation, head-loading is the most common method, and there is a significant MSK health burden [91]. One study reports that water is the most frequently carried material, but it ranks behind firewood and agricultural items in terms of difficulty and duration of loading [44]. Whereas, another study reports that water is more difficult to carry due to the unstable, ‘sloshing’ nature of transporting water compared to stable firewood [43]. These findings highlight the need to account for such confounding or compounding experiences that may influence other studies’ findings on the relationship between water fetching and MSK health. This paper does not assume that water carrying is the only nor the greatest contributor to MSK loading, but it focuses specifically on the loading of water to highlight how improved water accessibility can alleviate some burden of physical injury and disability in low-resource settings.

Lastly, by limiting the search criteria to SSA and only including English papers, research on the MSK health of water fetchers from other countries or from authors of different languages has not been included. This may have resulted in not identifying all papers that assess the MSK health of water fetchers, which could potentially provide data applicable to the SSA populations who carry water similarly.

## Conclusion

The reported MSK pain, injuries, and disabilities in this review are preventable, and this is an important global public health problem that is socially normalized, under-measured, and underrepresented in the literature. Water is a human right and the distance travelled to water increases the risk of MSK injury throughout the water fetcher’s life via various acute and chronic mechanisms. This is a gendered issue that disproportionately impacts women, and it warrants greater research, policies, innovations, and healthcare services. Unpaid domestic labour such as water fetching is crucial for the functioning of a household and ultimately for national and global economies. African women are key players in the social progress, economic development, and climate resiliency needed in the decades to come. This research contributes to an *academic movement* that supports women’s *physical movements*, and therefore, women’s health and equity.

## Supporting information

S1 Table Ovid Medline search strategy (n = 1344) [Searched March 15, 2023]. (TIF)

S2 Table Ovid Embase search strategy (n = 692) [Searched March 15, 2023]. (TIF)

S3 Table CINHAL search strategy (n = 439) [Searched March 15, 2023]. (TIF)

S4 Table Grey literature database searching. (TIF)

S5 Table Customized Google searching. (TIF)

S6 Table Targeted website searching. (TIF)

S7 Table Consultation with experts in field of study. (TIF)

S8 Table All studies identified in literature search. (XLS)
