# Supplementary figures and images for: Water fetching and musculoskeletal health across the life-course in Sub-Saharan Africa: A scoping review
Source: PLOS Glob Public Health. 2024 Sep 3;4(9):e0003630. doi: 10.1371/journal.pgph.0003630 (PMC11371245; doi:10.1371/journal.pgph.0003630)

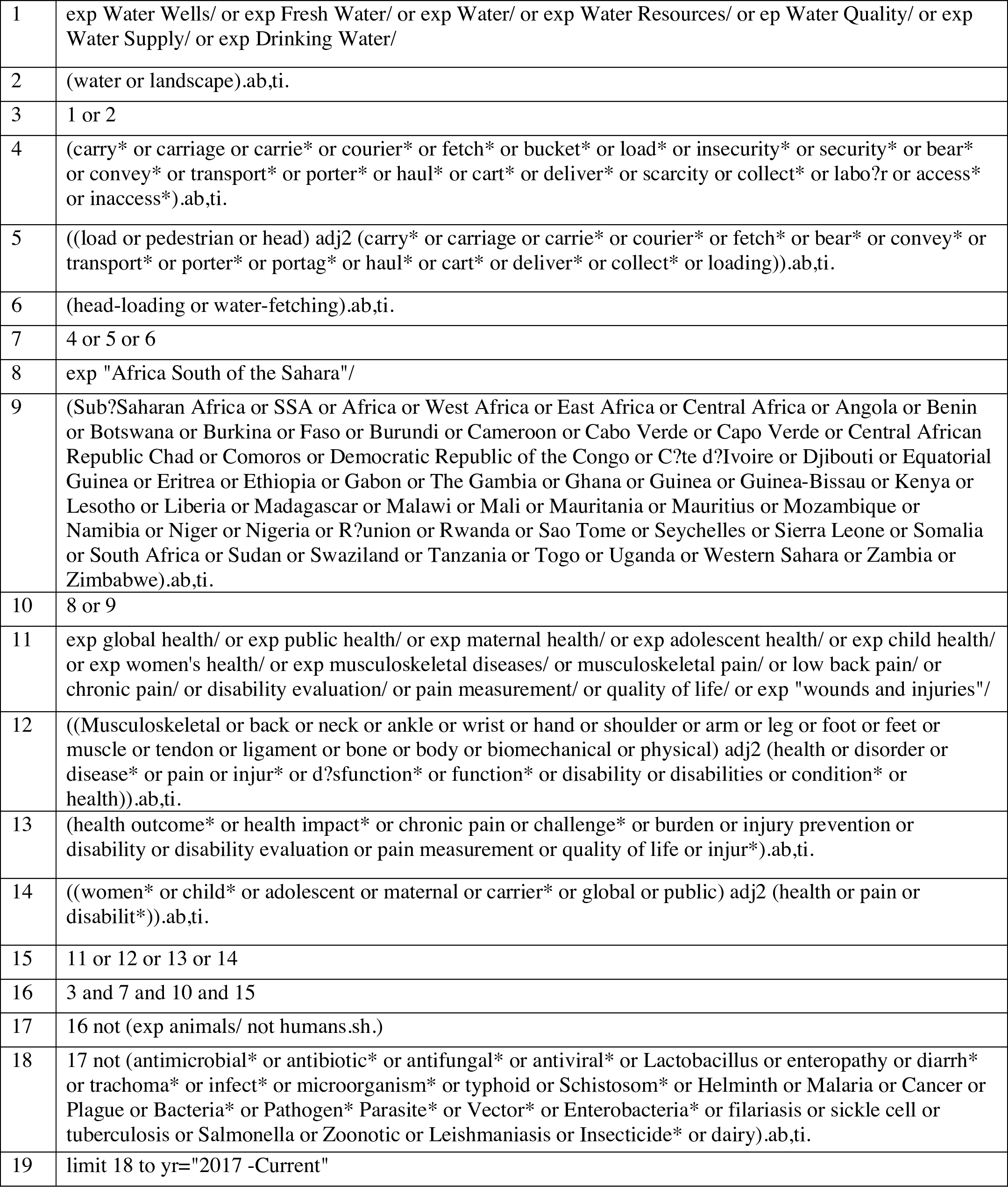

Supplement: S1 Table — (TIF) [file pgph.0003630.s001.tif]

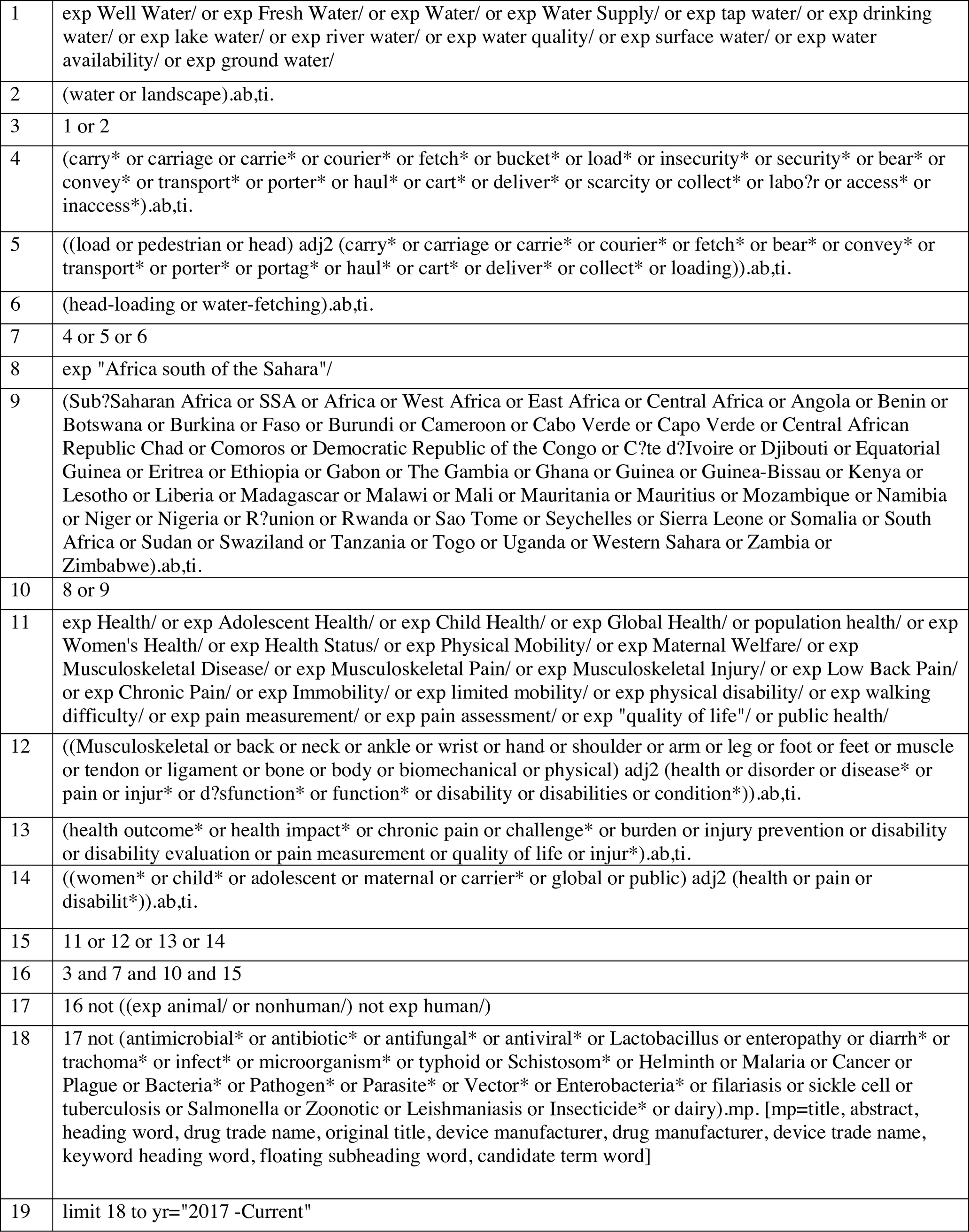

Supplement: S2 Table — (TIF) [file pgph.0003630.s002.tif]

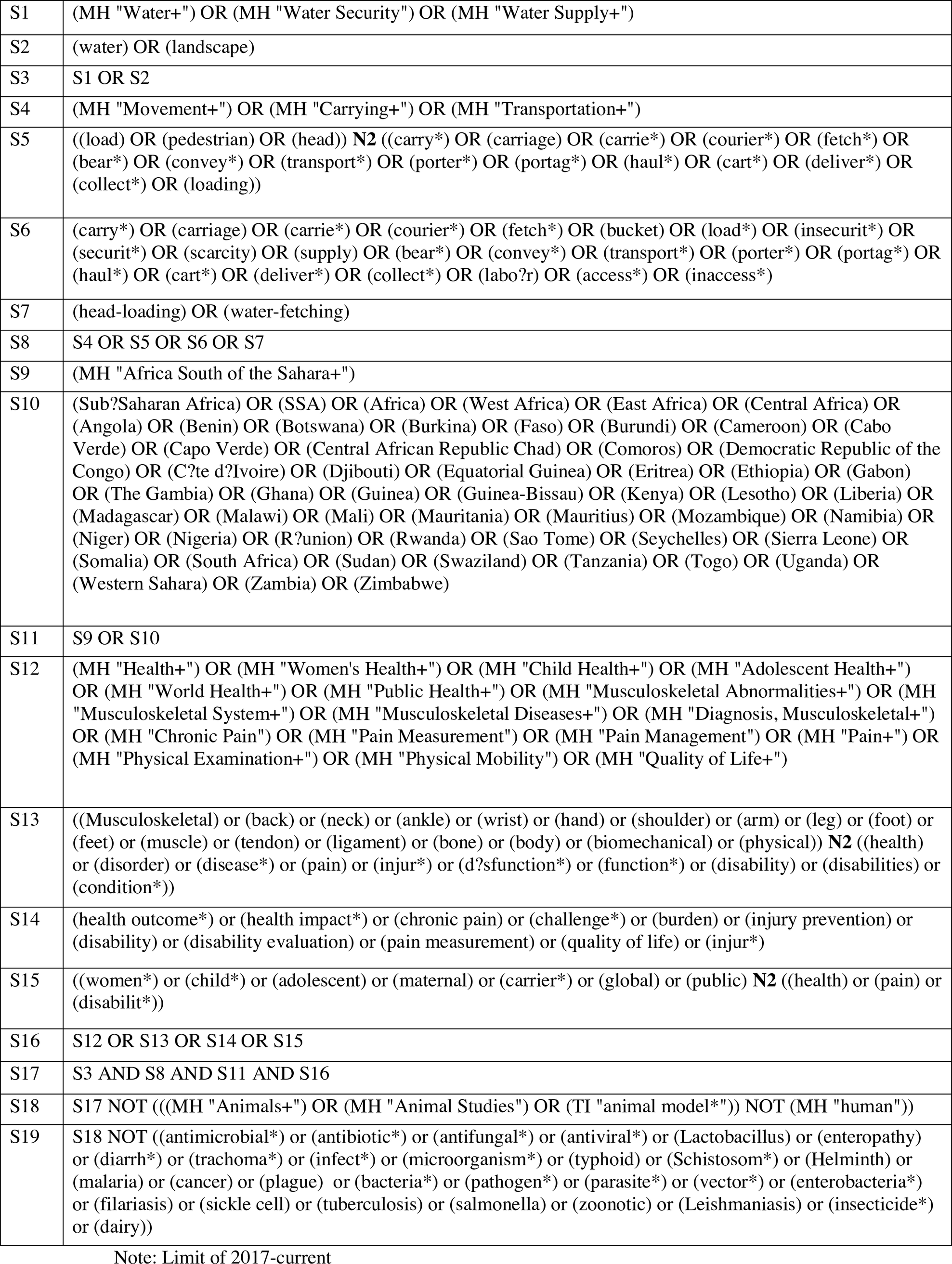

Supplement: S3 Table — (TIF) [file pgph.0003630.s003.tif]

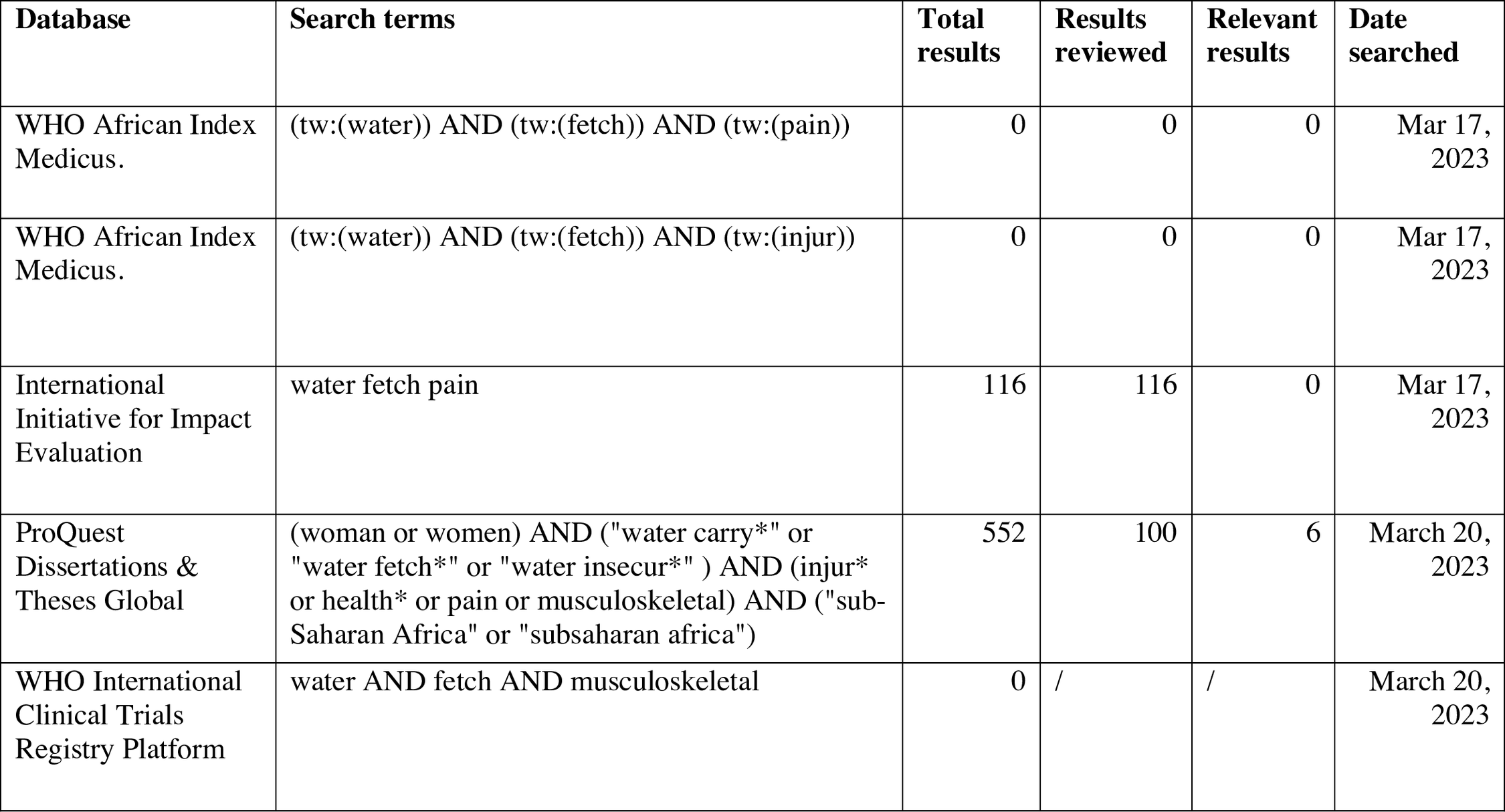

Supplement: S4 Table — (TIF) [file pgph.0003630.s004.tif]

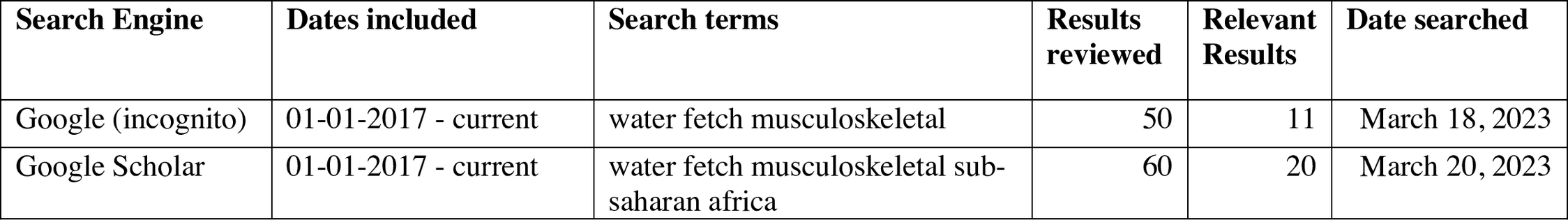

Supplement: S5 Table — (TIF) [file pgph.0003630.s005.tif]

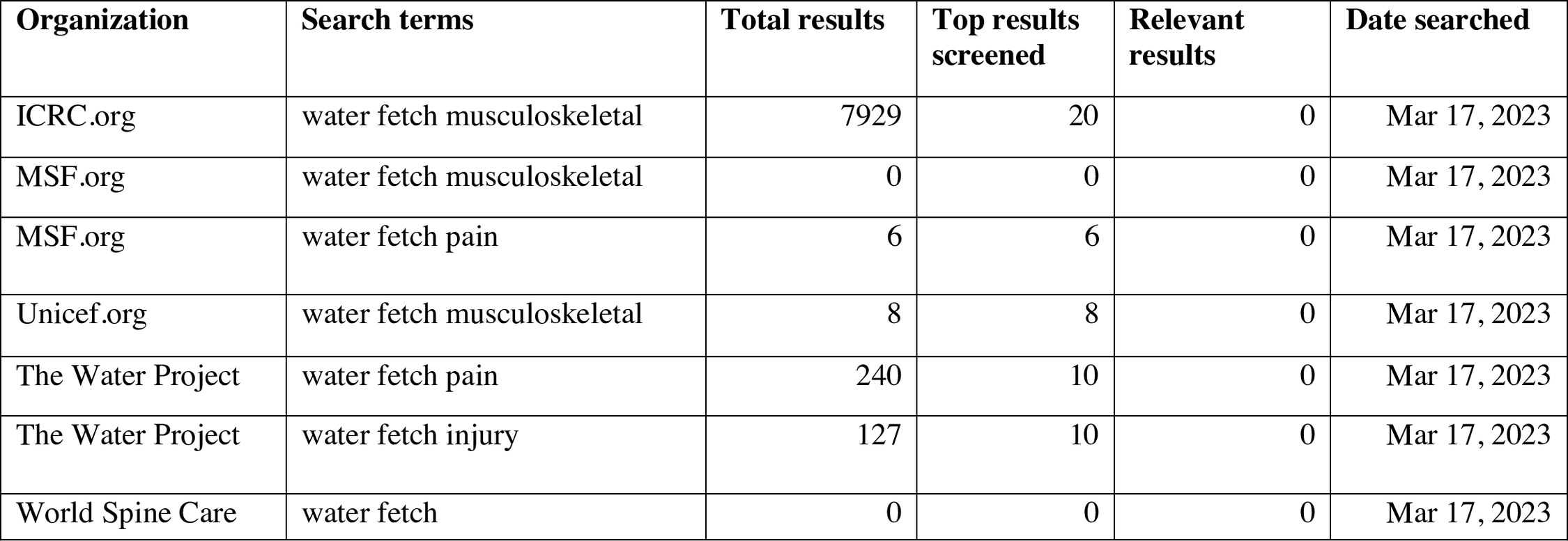

Supplement: S6 Table — (TIF) [file pgph.0003630.s006.tif]

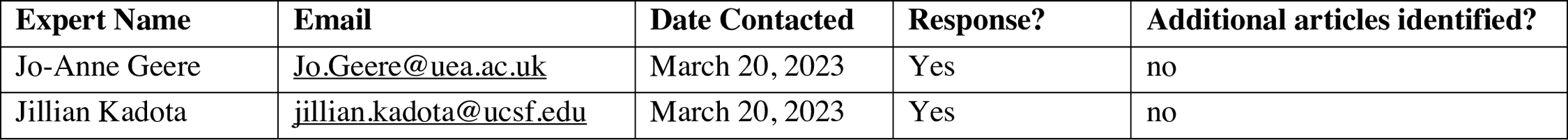

Supplement: S7 Table — (TIF) [file pgph.0003630.s007.tif]
